# Mechanical and Morphological Properties of Polypropylene/Nano ****α****-Al_**2**_O_**3**_ Composites

**DOI:** 10.1155/2014/718765

**Published:** 2014-02-03

**Authors:** F. Mirjalili, L. Chuah, E. Salahi

**Affiliations:** ^1^Department of Material Engineering, Maybod Branch, Islamic Azad University, Maybod, Iran; ^2^Department of Chemical & Environmental Engineering, Faculty of Engineering, Universiti Putra Malaysia (UPM), 43400 Serdang, Selangor, Malaysia; ^3^Ceramic Department, Material and Energy Research Center, Meshkin dasht, Karaj, Tehran 31787-316, Iran

## Abstract

A nanocomposite containing polypropylene (PP) and nano **α**-Al_2_O_3_ particles was prepared using a Haake internal mixer. Mechanical tests, such as tensile and flexural tests, showed that mechanical properties of the composite were enhanced by addition of nano **α**-Al_2_O_3_ particles and dispersant agent to the polymer. Tensile strength was approximately ∼16% higher than pure PP by increasing the nano **α**-Al_2_O_3_ loading from 1 to 4 wt% into the PP matrix. The results of flexural analysis indicated that the maximum values of flexural strength and flexural modulus for nanocomposite without dispersant were 50.5 and 1954 MPa and for nanocomposite with dispersant were 55.88 MPa and 2818 MPa, respectively. However, higher concentration of nano **α**-Al_2_O_3_ loading resulted in reduction of those mechanical properties that could be due to agglomeration of nano **α**-Al_2_O_3_ particles. Transmission and scanning electron microscopic observations of the nanocomposites also showed that fracture surface became rougher by increasing the content of filler loading from 1 to 4% wt.

## 1. Introduction

Polymeric materials are, fundamentally, devised for definite relevancies because of their construction and properties. However, a polymer needs some modifications in its structure or physical properties to obtain a superior range of functions. One modification technique is adding fillers to a polymer to generate a composite with improved properties, such as enhancement in mechanical strength, electrical conductivity, or thermal stability. The utilization of nanoscale fillers in particular has led to a significant improvement in the properties of the polymer [1–3]. Polymeric composite in which one of the constituents has dimension of 1–100 nm is called polymer nanocomposite or nanocomposite. The nanoscale fillers are used as reinforcement materials for nanocomposites containing nanoparticles, nanofibers, nanoplatelets, nanoclays, and so forth. These nanocomposites can be broadly used in diverse fields such as plastics, rubbers, coatings, and inks.

Nanocomposites exhibited superior mechanical performance and improved barrier properties at much lower loading levels of nanofiller compared to usual fillers. However, strong chemical bonding between the nanofillers and the polymeric matrix is compulsory in order to produce a nanocomposite with excellent mechanical properties [4].

The other key issues in preparation of nanocomposites are uniform dispersion of nanofillers against their agglomeration due to van der Waals's bonding alignment of nanoparticles in the matrix and proper selection of nanofiller/matrix volume fraction. Besides those key issues, manufacturing rate and cost-effectiveness of nanocomposite synthesis need to be well considered. In order to improve dispersion of nanofillers, there are at least two ways in practice; the first one is to treat the surface of nanofillers and the second one is to modify the surface of polymeric matrix [5]. Polymeric nanocomposites that possess increased stiffness and ductility with having a reasonably low loading of nanofillers were reported [3]. The enhancements are normally established by a few factors, such as the inherent features of nanofillers and polymers, the interface and the interphase among the constituent components, and dispersion of nanofillers in the matrix [6]. Nanocomposites produced using either organic or inorganic nanofillers have been the main focus of numerous researches in current years as they generally not only dwell in the merged properties of organic polymers (e.g., elasticity, ductility, and dielectric) and inorganic materials (e.g., high thermal permanence, strength, hardness, and high refractive catalog), but also they may obtain some particular or new properties. Besides their developed properties, another advantage of using nanoscale reinforcements is having the capability of developed process and being recyclable [7]. Among different types of polymers used, polypropylene is a semicrystalline thermoplastic that is characterized by light weight, low cost, easy processing, high mechanical strength, excellent chemical stability, and electrical properties [4, 8]. Alumina is amongst the normally used mineral particles in composite industry. However, there is little research on the Al_2_O_3_/PP nanocomposite, and therefore current study will set the concentration on this sort of nanocomposite system. Additional reason for selecting alumina nanoparticles is that the spherical nanoparticle shape of alumina will create a simple composite system which lacks orientation effect. With the rapid development of nanotechnologies and nanomaterials since 1990s, studies on polymer-based nanocomposites have been extensively carried out in order to find promising alternatives to traditional composites, while this study mainly focuses on providing the comprehensive differences between general mechanical and morphological properties of pure PP and PP/nano *α*-Al_2_O_3_ composites as well as surface modification of nanoparticles to produce better dispersions in organic solvents. The novelty of current study is the use of SDBS as a dispersant through ultrasonication method to disperse nanofiller extensively which will be mentioned below. In the case of PP nanocomposites, the used filler is nanoalumina while titanium oxide (TiO_2_) is a coupling agent.

## 2. Materials and Methods

### 2.1. Materials

The PP grade 600G (melting temperature and melt flow rate are 165°C and 11 g/min and its density is 900 kg/m^3^) used in this work is supplied by Petronas Polymers Marketing and Trading Division Malaysia. Nano *α*-Al_2_O_3_ with the average particle size of 20–30 nm and density of 3106 kg/m^3^ is used. These nanoparticles are made in the laboratory by the procedure discussed by Mirjalili et al. [9–11]. Titanium dioxide powder with a minimum assay of 98% used as a coupling agent is supplied from Fisher Chemicals Sdn. Bhd., Malaysia. Sodium dodecylbenzene sulfonate (SDBS) purchased from Merck (Germany) is used as a dispersant.

### 2.2. Surface Treatment of Nano *α*-Al_2_O_3_

Ultrasonication (KQ2200DE Ultrasonic Cleanser, 100 W, Kunshan of Jiangsu Equipment Company, China) is used for preparation of miscellaneous aqueous nanosuspensions, as a conventional method for dispersing the extremely entwined or aggregated nanoparticle samples. About 0.1 g nano *α*-Al_2_O_3_ and water solution (99.8 g) with an anion surfactant (0.1 g SDBS) are mixed in a 150 mL beaker. The suspension is then sonicated for 1 h and then finally dried at 80°C for 4 hours [9]. In the nano *α*-Al_2_O_3_ suspensions, the optimized concentration for SDBS, which has the greatest dispersion results, is 0.1 wt%. This is in consistence with Zhu et al. [12] results in which they found that SDBS can partially ionize in water and give anionic species, while the boehmite holds positive charges in a neural aqueous medium, showing a strong affinity for anionic groups. The negatively charged dissociated species from surfactants are adsorbed on the positively charged boehmite surface and consequently the surface is negatively charged owing to the ionization of SDBS; hence, the effect of electrostatic stabilization is achieved. When more SDBS is added into the suspension, the concentration of Na^+^ group entering into the absorbed layer reduces the net charge of the powder surface which leads to a weak dispersion system.

### 2.3. Preparation of PP/Nano *α*-Al_2_O_3_ Composites Samples

In this investigation, spherical nano *α*-Al_2_O_3_ used to reinforce PP has the average particle size of 20–30 nm. Predrying process is carried out on the nano *α*-Al_2_O_3_ at 80°C for 4 hours in the controlled environment of a drying oven, to deliberately expel any moisture trapped inside the nano *α*-Al_2_O_3_ filler [13]. According to Akil et al. [14] the optimal content of coupling agent (TiO_2_) is 2 wt% of the filler. PP/nano *α*-Al_2_O_3_ composites containing 1, 2, 3, 4, and 5.0 wt% of fillers, respectively, are prepared for investigation. In this report, titanium oxide (TiO_2_) is used as a coupling agent on the surface of nanoalumina fillers in order to improve the addition of organic-inorganic interface. PP, nano *α*-Al_2_O_3 _and titanium oxide were weighted as listed in the Table 1.

**Figure 1:**
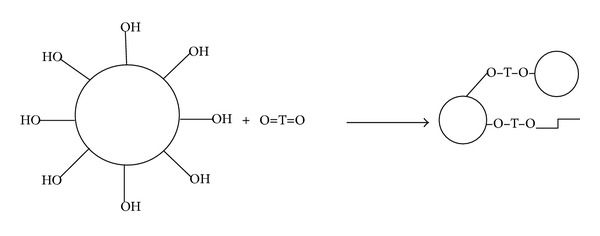
Schematic illustration of the reaction between titanium oxide and the hydroxyl groups of nanoalumina.

In general, titanium oxide can be represented as O=T=O and this unsaturated characteristic is expected to react with hydroxyl groups on the surface of inorganic fillers to give hydrophobic polymer-compatible monomolecular layers. In this manner, they promote adhesion, improve dispersion, lower viscosity, and also prevent phase separation [13–15]. The introduction of active groups onto nanoalumina surfaces is achieved by the reaction shown in Figure 1.

#### 2.3.1. Compounding and Processing

The melt blending of PP and nano *α*-Al_2_O_3_ powder is carried out using Thermo Haake Poly Drive with Rheomix R600/610 blending machine at 175°C with rotor speed of 50 rotational per minute (rpm). The first step of mixing involves the PP preheating for 4 min. After the preheating, the speed is preserved at 50 rpm for another 8 min of processing time to make sure of uniform heat distribution throughout the batch. Then, nano *α*-Al_2_O_3_ filler is included and titanium dioxide powder is added 2 minutes later. The rotor is stopped at the 12th minute and the melted compound is taken out for sheeting [13]. The melted compound of PP/nano *α*-Al_2_O_3_ is then formed by HSINCHU hot press machine, in the size of 15 × 15 cm. The compound is preheated for 2.5 minutes and hot pressed for 3 minutes, under the pressure of 150 kg/cm^2^ at 180°C, with 10 times of compression bumping. The sheet obtained is directly cooled with the cold press for 2.5 min of cooling cycle.

### 2.4. Characterization

#### 2.4.1. Tensile Tests

Tensile tests were carried out in accordance with ASTM D 638 using Instron Universal Testing Machine 4302. A load capacity of 1 KN at the crosshead speed of 1 mm/min was used. Specimens were formed into dumbbell shape and cut from the 1 mm compression molded sheet with a Wallace die cutter.

#### 2.4.2. Flexural Tests

Flexural tests were performed according to the ASTM D790-98 using Instron Universal Testing Machine (Model 3365), with a load cell of 5 KN. The test process used was three-point loading system utilizing center loading. The crosshead speed and span length were set to 3 mm/minute and 70 mm, respectively. The specimens were cut in to rectangular sizes with 3 mm thickness, 12 mm width, and 124 mm length.

#### 2.4.3. Dynamic Mechanical Analysis

The viscoelastic properties were measured by dynamic mechanical analyzer model PerkinElmer TE. The samples from compression molded plaques were cut into rectangular shape with the dimension of ~60 mm in length, ~13 mm in width, and ~3 mm in thickness. The measurement was carried out using three points bending flexural test. The samples were subjected to an oscillating frequency of 1 Hz and oscillating amplitude of 10 *μ*m in the temperature ranges of −30°C to 80°C at the heating rate of 2°C/min.

#### 2.4.4. Scanning Electron Microscopy (SEM)

Microscopic observation throughout the scanning electron microscopy (SEM) was achieved using Philips XL 30 ESEM operated at 20 to 30 kV.

#### 2.4.5. Transmission Electron Microscopy (TEM)

Transmission electron microscopy (TEM) observation was carried out on the ultrathin section of the PP/nano *α*-Al_2_O_3_ composite films to confirm the dispersion state of nano *α*-Al_2_O_3_ particles inserted within the PP matrix. The observation was conducted using a HITACHIA-7100 STEM at an acceleration voltage of 120 kV. The specimens were organized by using a Leica ultracut microtome provided with a cyrochamber.

## 3. Results and Discussion

### 3.1. Mechanical Properties

Mechanical properties of composites represent essential parts of their physical and chemical properties that are considered for most requests. Therefore, it is important to have basic information about mechanical performance of the composites and how this performance can be modified by the structural aspects which can be differing in polymers. It is recognized that the mechanical properties of particulate filled polymer composites depend on high amount of interfacial adhesion and filler dispersion [15]. In this study, titanium oxide (TiO_2_) was used as a coupling agent on the surface of nano *α*-Al_2_O_3_ fillers. Titanium oxide may increase the nucleating ability of the fillers and retard the motion of the PP chains. This leads to the formation of spheroid PP crystals which are less perfect and smaller in size around the particles in the matrix and promote plastic deformation of the matrix after the debonding occurs. Moreover, this incident prevents crazing of the polymeric matrix and allows for extensive plastic deformation, resulting in large quantities of fracture energy [5, 13, 14].

#### 3.1.1. Tensile Tests

In general, properties obtained from tensile test consist of the ultimate tensile strength, tensile modulus, and elongation at break. Determining of tensile strength and other tensile properties is important for evaluation of different polymers and component designs, design of plastic components for load-bearing applications, and, finally, for determining the specifications and predicting in-service performance of plastics.

**Figure 2:**
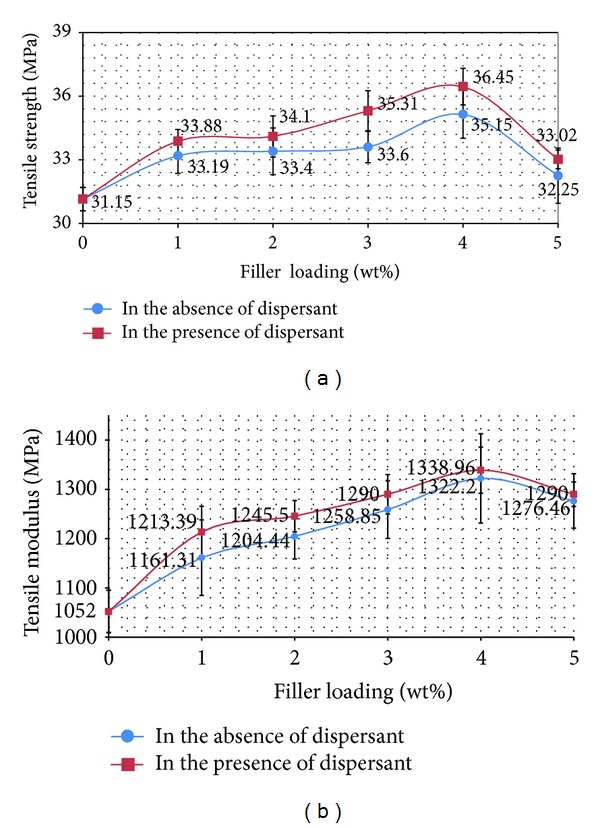
Tensile strength and tensile modulus of PP/nano *α*-Al_2_O_3_ composites at various loadings.

*(1) Tensile Strength and Tensile Modulus.* Figure 2 presents the tensile properties of the virgin PP and the Al_2_O_3_/PP nanocomposites. As it is shown in Figures 2(a) and 2(b), both the tensile modulus and tensile yield strength values are slightly increased as a function of nanoparticle content.

It proposes that a stiffness enhancement effect by the addition of the Al_2_O_3_ nanoparticles is significant. As a result of the enormous specific surface area of the nanoparticles, in spite of a very low particle loading, the total interface area or interface zone (IZ) is larger than that of a conventional microparticulate composite. Therefore, the property of IZ plays a main role in determining the property of the nanocomposite, in addition to the particle dispersion. Conversely, titanium oxide coupling agents have been broadly used in the modification of ceramic particles in researches on PP composites. It is reported that the interfacial interaction can be improved between the particles and the PP matrix. In the presence of strong interfacial interaction, the load transfer across the nanoparticle-matrix interface takes place very easily, which provides the raise in the tensile modulus. This observation, which is in accordance with the findings reported by Akil et al. [14], can be explained by the fact that the fillers have higher stiffness than the matrix and are able to improve the modulus of the composite. Regularly, the ability of composite interface to transmit elastic deformation depends upon the interfacial stiffness and static adhesion strength. A higher interfacial stiffness corresponds to a high composite modulus. Secondly, the tensile yield strength of the nanocomposite from Figure 2(a) also shows a minor increasing trend compared with that of the virgin PP. For the nanocomposites, if there is some deal of particle debonding from the matrix happening prior to the obvious plastic deformation of the matrix, generally the yield strength will reduce. Hence, the improvement in the tensile yield strength supplies a proof that the interaction between the nanoparticles and PP matrix is enough to restrict the considerable matrix yielding. As it can be seen in Figures 2(a) and 2(b), after the formation of some aggregates, these properties are decreased. This is directly related to the microstructure of nanocomposites because the size of aggregates increases by increasing the nano *α*-Al_2_O_3_ content [5]. At low nano *α*-Al_2_O_3_ content nanofillers act as reinforcement agents, but at higher contents the formed aggregates act as mechanical failure concentrators. Thus, PP nanocomposites exhibit their higher mechanical performance at concentration 4 wt% while, with further increase of nano *α*-Al_2_O_3_, the tensile strength of PP/nano *α*-Al_2_O_3_ composites decreases. At low nano *α*-Al_2_O_3_ content, partial tensile strain can be transferred to nano *α*-Al_2_O_3_s embedded in PP matrix under tensile stress, which leads to the increase of tensile strength. With further addition of nano *α*-Al_2_O_3_s, more agglomerates of nano *α*-Al_2_O_3_s form in PP matrix and many defects are introduced into the polymer matrix due to the difficulty of homogeneously dispersing nano *α*-Al_2_O_3_s by melt mixing. These defects lead to the decrease of tensile strength. However, in all the cases the mechanical properties are higher than pure PP indicating the reinforcement effect of nano *α*-Al_2_O_3_s on PP matrix. This is consistent with Bikiaris et al. [16] studies in which it is explained that the formation of agglomeration is justification for decreasing the mechanical properties especially when the size of aggregates is increased by adding the nanofillers content.

Moreover, Khalid et al. [17] and Mahfuz et al. [18] presented the creation of filler agglomeration site inside the matrix body that possibly acts as the failure initiation sites, which might help in the propagation of the crack or fracture. This problem becomes more critical when the content of nanofiller is too high.

The effect of dispersant (SDBS) on tensile strength of PP/nano *α*-Al_2_O_3_ composite is also shown in Figure 1. As it is noted, dispersant improved the tensile strength and tensile modulus of the nanocomposite, compared to those samples without SDBS. PP/nano *α*-Al_2_O_3_ composite prepared using filler with the amount of up to 4 wt% and dispersant showed the highest value of tensile strength and tensile modulus, which is up to 36.45 MPa and 1338.96 MPa, respectively. The reasons for this phenomenon are well explained by Huang et al. [5]. They stated that SDBS acts like a surfactant and breaks up the large agglomerates of nano *α*-Al_2_O_3_ particles into fine ones. Moreover, it may cause good wet ability of the nano *α*-Al_2_O_3_ particles by PP and hence the tensile properties are increased. 

**Figure 3:**
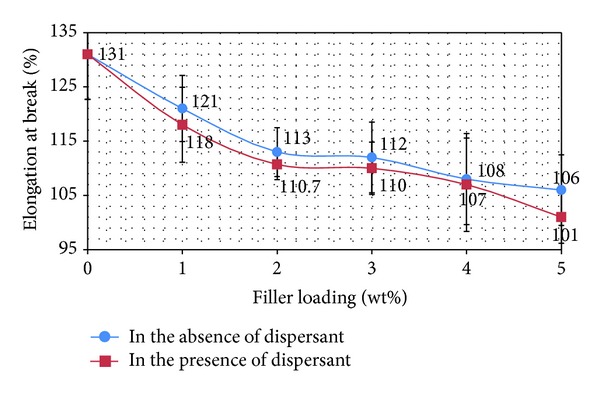
Elongation at break of PP/nano *α*-Al_2_O_3_ composites at various loadings.

*(2) Elongation at Break of PP/Nano *α*-Al*_*2*_ *O*_*3*_ *Composites*. From an engineering point of view, elongation at break is a significant factor recounting the rupture performance of composites [19]. Elongation at break for PP/nano *α*-Al_2_O_3_ composites was considerably decreased with increasing of filler loading as it is shown in Figure 3.

As the elongation is reciprocal of the stiffness of a material, the results show that the filler imparts a greater stiffening effect [4, 20]. It can be seen that by addition of 5 wt% *α*-Al_2_O_3_ nanoparticles, elongation is drastically dropped. The percentage of elongation at break is approximately 22% to 30%. The dramatic reduction in elongation at break implied that the ductility of PP has been suppressed by the presence of nano Al_2_O_3_ particles. Comparison of PP/nano *α*-Al_2_O_3_ composites in the absence and presence of dispersant (Figure 2) indicates that the nanocomposite prepared in the presence of dispersant has lower value for elongation at break than the nanocomposite prepared in the absence of the dispersant.

#### 3.1.2. Flexural Tests

Flexural properties are considered when the maximum stress and strain occur outside the surface of the test bar [21]. Flexural tests have more advantages than tensile tests. For instance, if a material is used in the type of a beam and if the service breakdown occurs in bending, then a flexural test is more important for designing than a tensile test. The flexural specimen is reasonably simple to organize without outstanding strain. The specimen configuration is also more difficult in tensile tests. Additional advantage of the flexural test is that, at small strains, the real deformation can be measured precisely [19, 21].

*(1) Flexural Strength and Flexural Modulus*. Flexural strength (FS) is the capacity of a material to resist the bending forces applied perpendicular to its longitudinal axis [22]. Figure 3 shows an improvement of flexural strength for nanocomposites versus filler loading. It is expected that the FS will be further increased with the addition of nano *α*-Al_2_O_3_ loading, if and only if the interfacial adhesion between nano Al_2_O_3_ and the polymer matrix is strong enough. The strength of interaction depends on the surface energy of the components. Silicates, aluminates, and titanates usually have high surface energy that leads to relatively strong interaction between the matrix and the filler. Rasheed et al. [22] also explained that the flexural properties are strongly affected by the quality of the interface in composites. The static adhesion strength, in addition to the interfacial stiffness, acts as a main function to increase the filler reinforcement.

The highest flexural strength was achieved at 55.88 MPa for a nanocomposite which contains 5 wt% nano *α*-Al_2_O_3_ particles, and dispersant was used for its preparation. It is expected that the flexural strength is further slightly enhanced with the addition of nano *α*-Al_2_O_3_ particles loading, since they are rigid materials, and, by increasing the amount of their loading, rigidity of the nanocomposites increased, and hence ductility decreased. Addition of 5 wt% nano *α*-Al_2_O_3_ particles into the PP matrix caused an increase in FS value of the nanocomposites. The FS value obtained for PP/nano *α*-Al_2_O_3_ composites in the presence of dispersant increased about 49% while this value for nanocomposite prepared in the absence of dispersant increased maximum 34.66% in comparison with the FS value of the virgin PP system.

**Figure 4:**
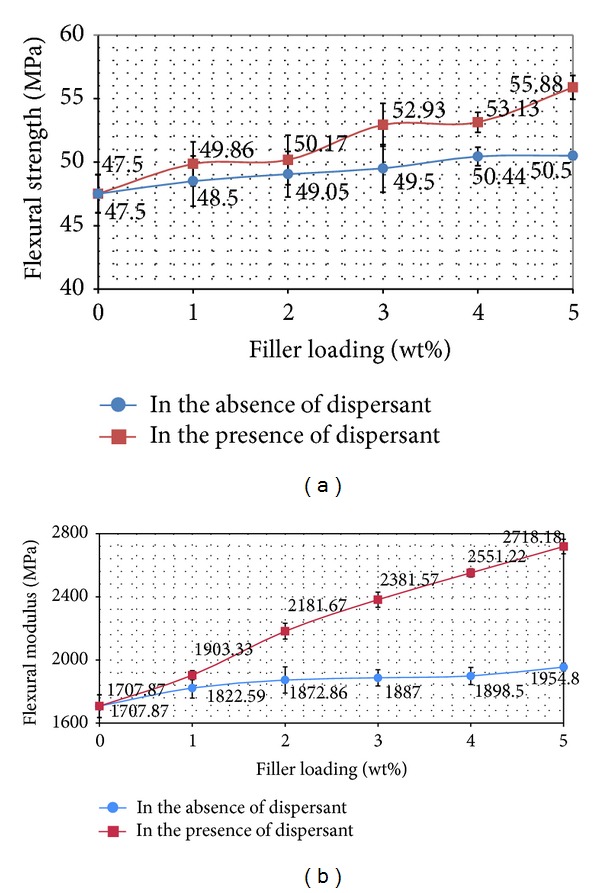
Flexural strength and flexural modulus of PP/nano *α*-Al_2_O_3_ composites at various loadings.

Flexural modulus (FM) reported in this section points out that the nanocomposites emulate the rigidity of fillers and change from the normally flexible plastic to stronger materials.  Figure 4(b) shows a pattern for nanocomposite indicating that nano *α*-Al_2_O_3_ particles cause the rising in flexural modulus, especially in the presence of dispersant. In general, when hard/rigid filler is integrated into the polymer matrix, the modulus will be enhanced [3, 23].

By addition of 5 wt% nano *α*-Al_2_O_3_ to the PP matrix, flexural modulus of the nanocomposites prepared using dispersant increased to 2718 MPa. Similarly, this value for nanocomposites prepared without using dispersant was 1954 MPa, which is higher than flexural modulus of pure PP. An improvement of about 60% was found in modulus value of the nanocomposites that dispersant was used in the preparation procedure. The improvement in modulus value of the nanocomposite decreased about 14.45% when no dispersant was used. Recall that SDBS improves dispersion of nano *α*-Al_2_O_3_ particles and the aggregates are shifted to the fine particles. It also increases the ability of nanocomposite interface to transmit elastic deformation that causes increasing of the flexural modulus values [23, 24].

### 3.2. Viscoelastic Properties

**Figure 5:**
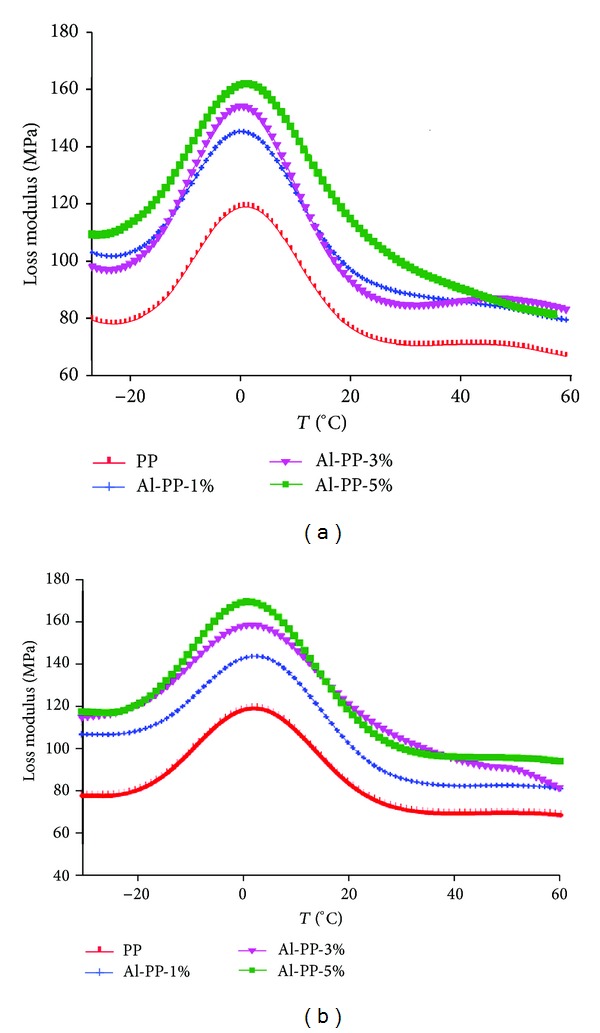
Loss modulus of unfilled PP and PP/nano *α*-Al_2_O_3_ composites with different filler loading (a) in the absence of dispersant and (b) in the presence of dispersant.

The effect of nano *α*-Al_2_O_3_ particles on the viscoelastic properties of polymers has been probed using DMA. This technique allows investigating the relaxation related to the Brownian motion of the main chains due to the transition occurring during the cure process. It is likely that this motion can be affected by nano *α*-Al_2_O_3_ particles because of interactions in the interface layer around the particles. Referring to Figure 5 the loss modulus of unfilled PP was improved with the addition of nano *α*-Al_2_O_3_ loading. It seems that a higher viscosity influences the molecular movement due to the existence of the fillers. Joseph et al. [24] reported that the existence of fillers causes the rising of the loss modulus that indicates that he higher viscosity because of the existence of the fillers leads to a restriction in molecular movement. The higher the nano *α*-Al_2_O_3_ content is, the higher the viscosity will be, which ultimately needs extra energy for dissipation. Based on Figure 5, in the case of pure PP, a loss modulus peak is observed around 5°C, which is supposed to be the *β*-transition. This transition did not change as the filler loading was increased. The *β*-transition, correlated with the glass-rubbery transition, is attributed to molecular motions related to unrestricted amorphous PP [14, 21, 24].

At the high concentration of fillers, dispersion of the filler into the polymer matrix is irregular and the void of the cross-linking increases. However, the main important factors that can affect the *β*-transition are degree of particle dispersion and curing conditions [13]. The nanoparticle agglomeration is pronounced as proved by SEM and TEM observations. These results confirmed the tensile and flexural results that the presence of nano *α*-Al_2_O_3_ particles increases the mechanical properties.

Based on Figures 5(a) and 5(b), treatment in the presence of SDBS as a dispersant increased the active surface area of the nano *α*-Al_2_O_3_, ensuing improvement in wetting of the nano *α*-Al_2_O_3_ during compounding, and, therefore, it causes a superbonding between filler and matrix.

### 3.3. Morphological Analysis of Nano *α*-Al_2_O_3_ Particles and PP/Nano *α*-Al_2_O_3_ Composites

To study the morphology and microstructure of the nanocomposites and nanoparticles, SEM and TEM were carried out. Scanning electron microscopy used a focused beam of high-energy electrons to generate a variety of signals at the surface of solid specimens [25]. Transmission electron microscopy is a microscopy technique where a beam of electrons is transmitted through an ultrathin specimen, interacting with the specimen as it passes through. Vladimirov et al. [4] found out that filler dispersion and adhesion with the polymer matrix are the basis for efficiently enhancing the mechanical behavior of composites. Fine control of the interface morphology of the nanocomposites is one of the most serious factors to convey the desired mechanical properties on such materials.

#### 3.3.1. Scanning Electron Microscopy (SEM) Analyses of Nano *α*-Al_2_O_3_ Particles

**Figure 6:**
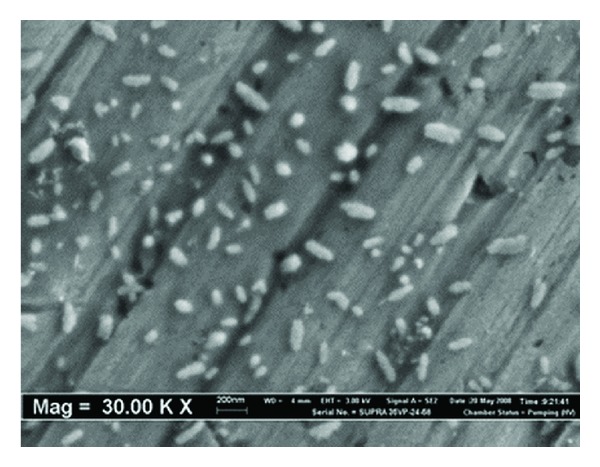
SEM micrograph of shape and size distribution of nano *α*-Al_2_O_3_ particles.

Figure 6 shows SEM image of the nano *α*-Al_2_O_3_ particles, from which it can be seen that most nanoparticles are spherical shape. The average diameter of the *α*-Al_2_O_3_ nanoparticles is in the range of 20–30 nm with the surface area of 412 m^2^ g^−1^ [9–11].

#### 3.3.2. SEM Analysis of Tensile Test Fractured Surface of PP/Nano *α*-Al_2_O_3_ Composites

**Figure 7:**
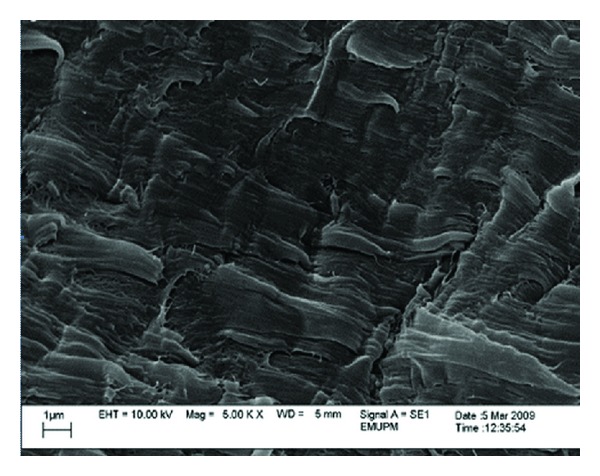
SEM micrograph of the fractured surface of pure PP sample.

SEM observations on fractured surfaces of the fabricated PP/nano *α*-Al_2_O_3_ composites were conducted to check the fracture behavior due to the tensile loading.  Figure 7 shows the SEM micrograph of the tensile fractured surface for pure PP samples. Pure PP has moderately smooth fractured surface and exhibits no symptom of plastic deformation or drawing. It is obvious that the comprehensive melting of PP was obtained while no void, hole, and impurities were detected. PP pellets were suitably bonded and well continued among each other. This situation produced excellent original strength to the matrix used, as completed by the outcomes of the tensile test.

**Figure 8:**
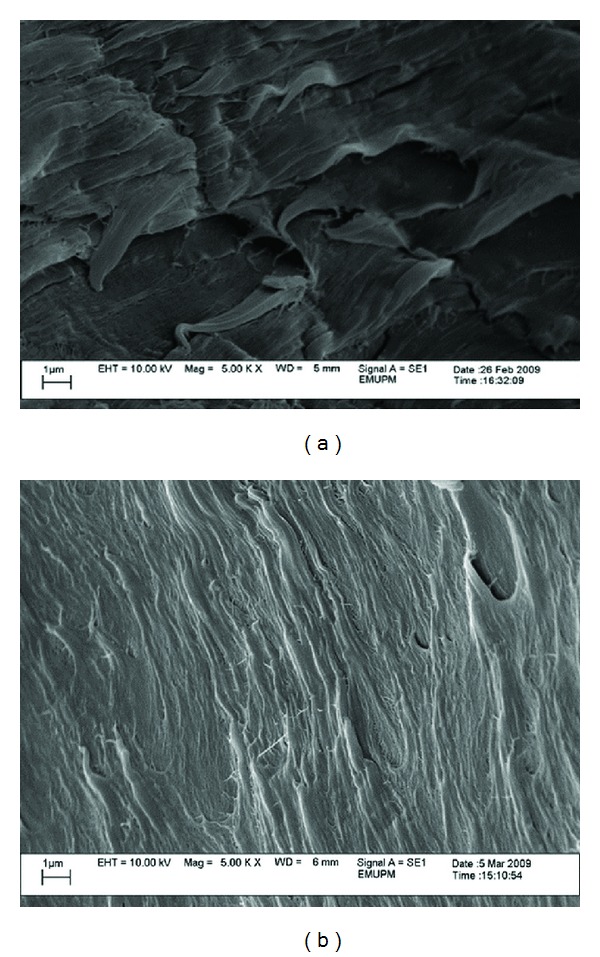
SEM micrograph of the fractured surface of PP/1% nano *α*-Al_2_O_3_ composites (a) in the presence of dispersant and (b) in the absence of dispersant.

**Figure 9:**
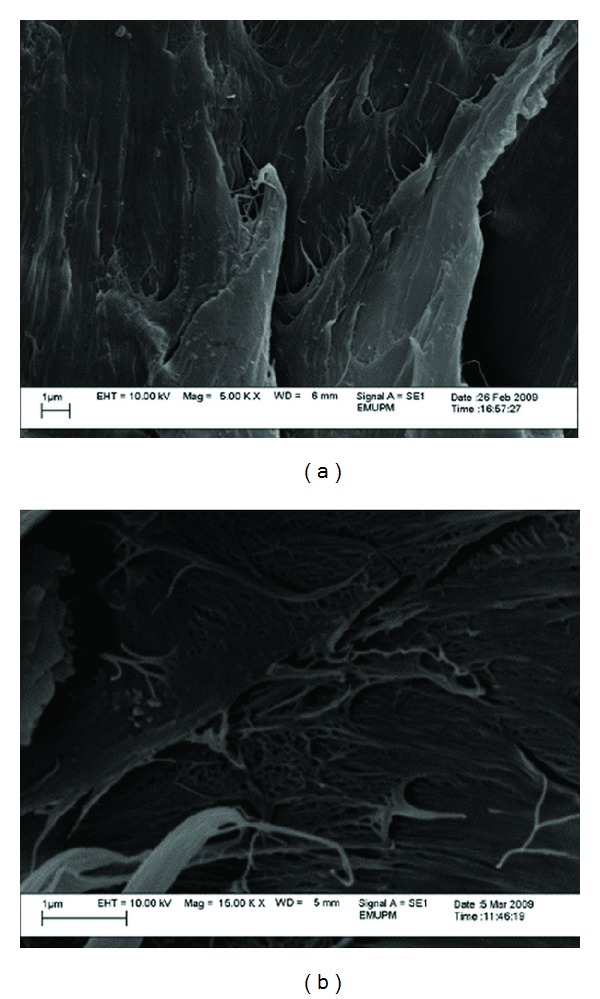
SEM micrograph of the fractured surface of PP/2% nano *α*-Al_2_O_3_ composites (a) in the presence of dispersant and (b) in the absence of dispersant.

**Figure 10:**
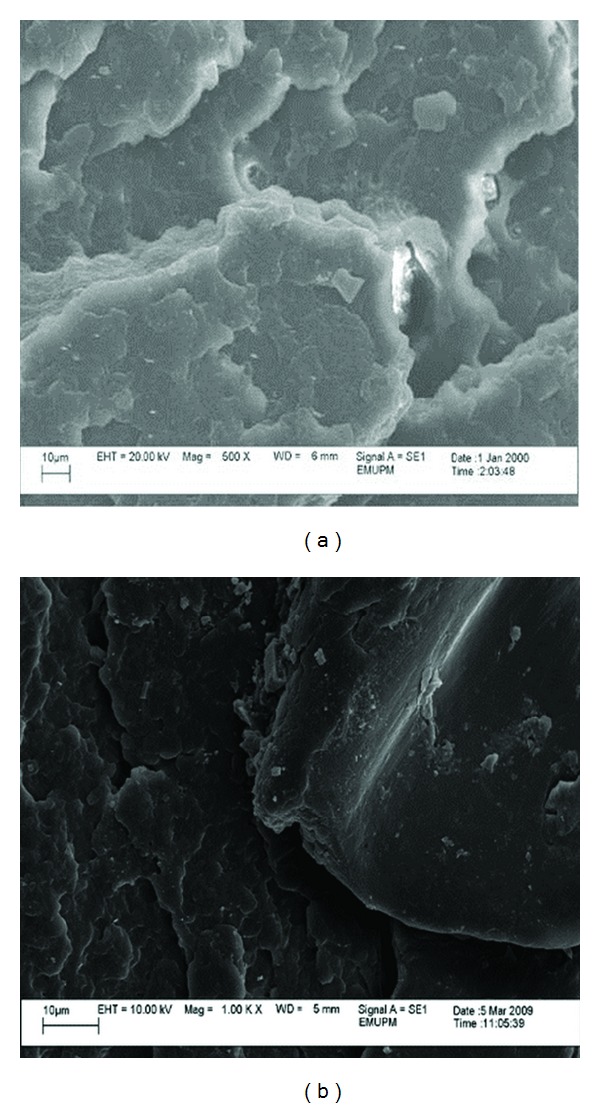
SEM micrograph of the fractured surface of PP/3% nano *α*-Al_2_O_3_ composites (a) in the presence of dispersant and (b) in the absence of dispersant.

**Figure 11:**
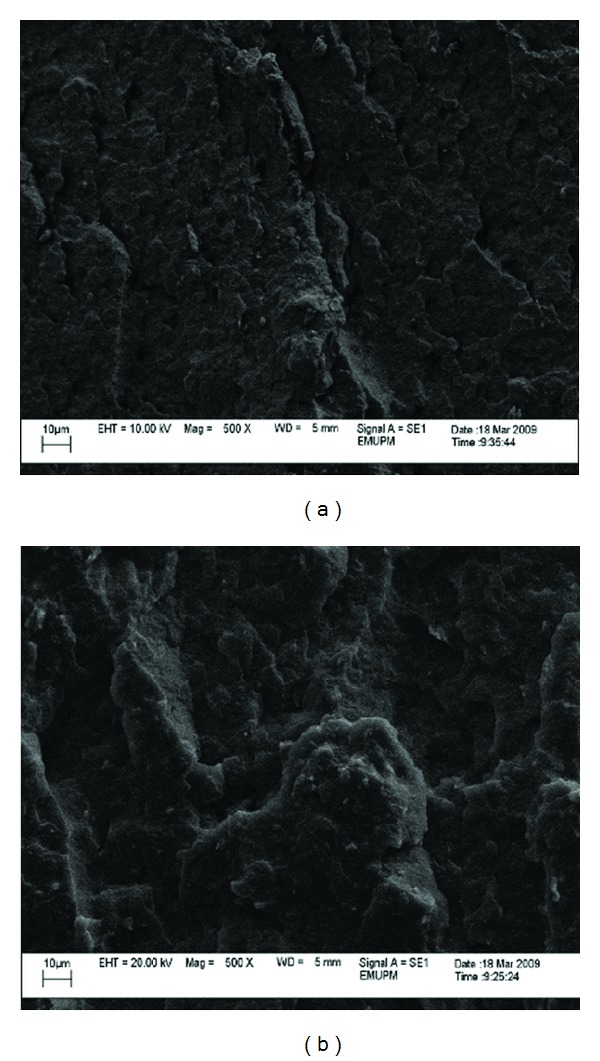
SEM micrograph of the fractured surface of PP/5% nano *α*-Al_2_O_3_ composites (a) in the presence of dispersant and (b) in the absence of dispersant.

From the micrographs in Figures 8, 9, 10, and 11, it can be seen that composites with different nano *α*-Al_2_O_3_ contents exhibited different nano *α*-Al_2_O_3_ dispersion state. At low nano *α*-Al_2_O_3_ content, most of the nano *α*-Al_2_O_3_s dispersed individually in the PP matrix as well as a good wetting behavior between the filler and matrix.

Good dispersion and wetting situations may generate desired stress distribution from the matrix to the filler throughout the tensile loading [19, 26, 27]. An increase in tensile strength after the addition of 1 wt% nano *α*-Al_2_O_3_ particles can be well represented by good dispersion and wetting situations as it can be observed in Figures 8(a) and 8(b).

In the case of PP/nano *α*-Al_2_O_3_ (2 wt%), the filler seemed to be embedded in the fracture surface of the nanocomposite. This is an indication of a good wetting behavior between the filler and matrix (Figure 9).

Addition of nano *α*-Al_2_O_3_ particles into PP matrix improved the rigidity of the fabricated nanocomposites and hence enhanced the strength of it. At the same time, ductility behavior totally reduced. At relatively high nano *α*-Al_2_O_3_ contents (3 wt%), nano *α*-Al_2_O_3_ aggregates appeared in the polymer matrix and the size of the aggregates increased with increasing nano *α*-Al_2_O_3_s content [10, 18]. Solid-state shear pulverization in conjunction with melt mixing was found to produce well-dispersed polymer/nano *α*-Al_2_O_3_s nanocomposites [28, 29].

As can be seen nano *α*-Al_2_O_3_s aggregates are observed as white spots with a wide particle size distribution. The particle sizes are mainly depended on the nano *α*-Al_2_O_3_s concentration. At low nano *α*-Al_2_O_3_ s content, nano particles formed agglomerates in lower magnitude which led to better dispersion. The mean particle sizes are in the range of 20 up to 30 nm, while as nano *α*-Al_2_O_3_s content increases nanoparticle forms larger agglomerates. This is due to the tendency of nanoparticles to interact with each other due to surface forces. These are very difficult to break down during nanocomposites fabrication, since the involved forces during melt extrusion are not enough to break these agglomerates.

Agglomeration and rough dispersion of nano *α*-Al_2_O_3_ particles can be clearly observed at higher filler loading. Therefore, a pitiable stress transfer from matrix to the filler is accounted for a decline in tensile properties with increasing of the filler loading. Tensile properties of the PP/nano *α*-Al_2_O_3_ also decreased as the arrangement of the voids will ease the propagation of stress. It is worth mentioning that the homogeneity of nano *α*-Al_2_O_3_ particles causes rough stress distribution during constant loading, and so early failure occurred that resulted in lower tensile properties. Morphology of the PP/nano *α*-Al_2_O_3_ composites with 5 wt% of the filler loading, shown in Figure 11, can be used to explain decreasing pattern of tensile strength of nanocomposites. Coleman et al. [28] reported that debonding will occur when the matrix fails under the large shear stress near the interface. This condition became more agglomerated and serious with the existence of large number of voids. These micrographs reveal that at higher filler loading, the nano *α*-Al_2_O_3_ particles tend to agglomerate, and therefore large holes or voids occur between nanoparticles and matrix. At 5 wt% nano *α*-Al_2_O_3_ particles content, extensive surface coarseness, in which the crack is improved, is seen. Impeded plastic deformation decreased tensile and flexural properties and failure strain. Interfacial adhesion became much weaker at higher concentration of nano *α*-Al_2_O_3_ owing to pitiable particle wetting by the matrix. This occurrence will permit the crack to broadcast at quicker rate (less adhesion), which affects the morphological suggestion exposed by the rougher surface as observed in Figure 11.

The results have proven that the interfacial adhesion of PP/*α*-Al_2_O_3_ nanocomposite prepared using dispersant can distribute the higher mechanical properties, because the aggregates are shifting to the primary particles during the ultrasonication process.

#### 3.3.3. TEM Analysis of PP/Nano *α*-Al_2_O_3_ Composites

**Figure 12:**
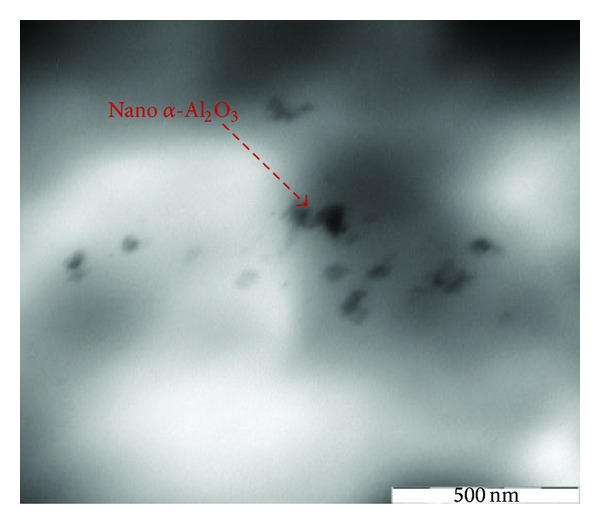
TEM observation of cryothin sectioning for PP/1% nano *α*-Al_2_O_3_ composites.

In order to explain the behaviors of the nanocomposites, the cryothin section of specimens was examined by TEM as illustrated in Figure 12. It can be seen that the particle dispersion of the 1 wt% PP/nano *α*-Al_2_O_3_ nanocomposite is pretty good. High magnification TEM micrographs of PP/1% *α*-Al_2_O_3_ nanocomposites show that the nano *α*-Al_2_O_3_ particles are close to spherical shape; although their sizes are not monodisperse, particles between the size of 27 and 35 nm are detected in the nanocomposite. These results are in good agreement with the Bikiaris et al. [16] and Zhao and Li [6] findings, in which the rise in the nano *α*-Al_2_O_3_ particles loading causes a little poorer dispersion of the nanoparticles but more agglomeration.

**Figure 13:**
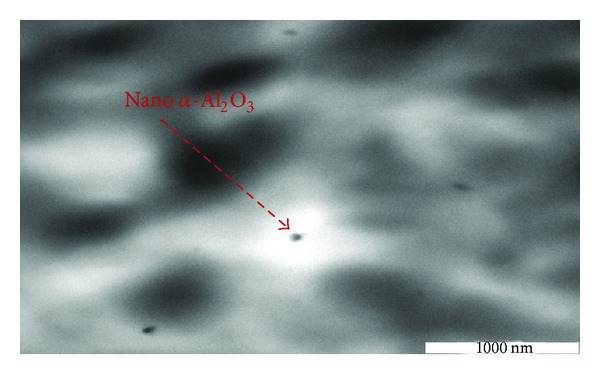
TEM observation of cryothin sectioning for PP/3% nano *α*-Al_2_O_3_ composites with dispersant.

Considering Figure 13 for those nanocomposites that contain dispersant with greater amounts of nano *α*-Al_2_O_3_ particles, up to 3, the size of nanoparticles is ranging between 50 and 60 nm. It is also obvious that the nano *α*-Al_2_O_3_ particles size slightly increased, and they were relativity well dispersed in the PP matrix as no significant amount of agglomeration is observed. Good and homogenous distribution of nano *α*-Al_2_O_3_ particles provide good stress shift throughout mechanical loading that ensures better mechanical properties of the manufactured composites [29, 30]. Therefore, this observation supports the positive results of mechanical properties testing, like tensile, flexural test, very well.

At low nano *α*-Al_2_O_3_ content, little agglomerations are also formed and it is impossible to disperse nanoparticles in their individual forms due to van der Waals's interactions, but most of the nano *α*-Al_2_O_3_s disperse individually in the PP matrix [30]. At relatively high nano *α*-Al_2_O_3_s contents, nano *α*-Al_2_O_3_s aggregates appear in the polymer matrix, and the size of the aggregates increases with increasing nano *α*-Al_2_O_3_s contents. This is due to the tendency of nanoparticles to interact with each other due to surface forces. These interact through weak van der Waals's forces creating clusters and agglomerates. These are very difficult to be broken down during nanocomposites fabrication, since the involved forces during melt extrusion are not enough to break these agglomerates [31], because during their mixture with PP, nano *α*-Al_2_O_3_ particles have a small tendency to be separated and dispersed homogenously into PP matrix at low nano *α*-Al_2_O_3_ particle concentration. This is due to the small interfacial adhesion between the nano *α*-Al_2_O_3_ particles and PP matrix.

**Figure 14:**
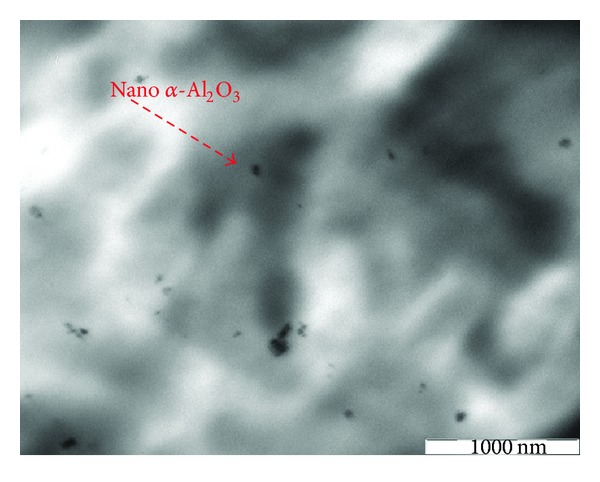
TEM observation of cryothin sectioning for PP/4% nano *α*-Al_2_O_3_ composites with dispersant.

However, a much higher degree of nano *α*-Al_2_O_3_ agglomerate exfoliation has been attained in PP/nano *α*-Al_2_O_3_s nanocomposites. PP-based nanocomposites exhibit strong filler aggregation, while well-separated individual nano *α*-Al_2_O_3_s prevail the use of PP/nano *α*-Al_2_O_3_s materials. In composition with more amounts of nano *α*-Al_2_O_3_ particles, up to 4 wt%, the sizes of nanoparticles range between 60 and 70 nm (Figure 14) and the formation of relatively compact nano *α*-Al_2_O_3_ particle takes place.

**Figure 15:**
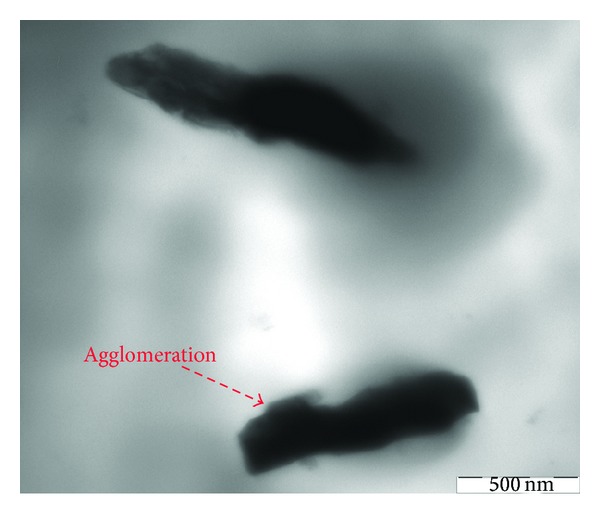
TEM observation of cryothin sectioning for PP/5% nano *α*-Al_2_O_3_ composites without dispersant.

**Figure 16:**
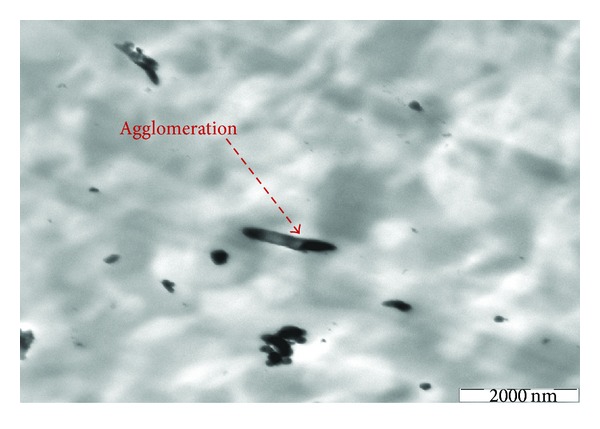
TEM observation of cryothin sectioning for PP/5% nano *α*-Al_2_O_3_ composites in the presence of dispersant.

At filler contents higher than 5 wt.% strongly agglomerated nano *α*-Al_2_O_3_ structures between 80 and 100 nm dimensions are formed in PP matrix. As Figures 15 and 16 show, higher concentrations of nano *α*-Al_2_O_3_ particles, up to 5 wt%, result in not well-dispersed individual nanoparticles, and even some nanoparticles are entangled together in the type of casual orientation, which generates the interconnecting structure. The size ranges of nano *α*-Al_2_O_3_ particles in the presence of dispersant and in the absence of dispersant were between 80–100 nm and 150–300 nm, respectively (Figures 15 and 16). Due to considerable nano *α*-Al_2_O_3_ aggregation, the PP/nano *α*-Al_2_O_3_s materials have substantially lower interfacial area in comparison with the respective PP/nano *α*-Al_2_O_3_s nanocomposites. Strikingly, when the nano *α*-Al_2_O_3_ contents are increased further to 5 wt%, the network density of fibrils is almost negligible, and instead crack growth on the matrix surface is spanned by nanoparticles bridging across the cracks assisted by network of fibrils and the crack toughness of the nanocomposite with 5 wt% of nano *α*-Al_2_O_3_s becomes a dynamic interplay of percolation and nanoparticles bridging leading to a semiductile behavior. Thus, the drastic hindrance of the ductile yielding in the polymer is controlled by the state of dispersion of the nano *α*-Al_2_O_3_s, the interface, and nano *α*-Al_2_O_3_s-induced structural reorganization. All the above-mentioned studies are consistent with Bikiaris et al. [16, 31] research which explains the microstructure of PP/nanofiller composites and reveals that nanofillers can form aggregates in PP matrix. These are very difficult to be destroyed during melt mixing while nanofillers dispersed intercalate into PP matrix. It seems that the applied shear forces are not strong enough to destroy the nanoparticle agglomerates. This microstructure directly affects most of the physical properties of PP/nanofiller composites and mainly the mechanical properties. At the low nanofiller concentration, a little enhancement is observed, while higher nanofiller concentrations cause deterioration. TEM joined with all the mechanical properties data supplies strong evidence that nano *α*-Al_2_O_3_ particle is the key to maximizing strengthening. For instance, tensile strength and tensile modulus are optimum at 4 wt% additions of nano *α*-Al_2_O_3_ particles, as discussed in this study.

The decrease in tensile strength after addition of 5 wt% of nano *α*-Al_2_O_3_ particles was validated from the observation of Figures 15 and 16.

From TEM micrographs, it was found that the presence of dispersant directly affected the size of the nanoparticles agglomerates. When SDBS is used, the nanoparticles agglomerates were broken up into stretched bonds [5, 25]. In this study, the agglomerate formation of nanoparticles is more serious, since the dispersion process was completely relying on the ability to separate the nano *α*-Al_2_O_3_ particles. At lower filler content, nano *α*-Al_2_O_3_ particles were dispersed very well, especially for the PP/nano *α*-Al_2_O_3_ composites with filler loading of lower than 5 wt% and increased the mechanical properties.

## 4. Conclusions

Polypropylene nanocomposites containing 1–5 wt% of nano *α*-Al_2_O_3_ particles were prepared using a Haake internal mixer in the present study. Dispersion of nano *α*-Al_2_O_3_ powders through the ultrasonication process with dispersion was investigated and used for preparing PP/nano *α*-Al_2_O_3_ composites. A comparison was made with PP/nano *α*-Al_2_O_3_ composites without dispersant. Tensile tests show that both Young's modulus and the tensile yield strength increase with the particle content up to 4% of nano *α*-Al_2_O_3_ filler adding. This suggests that the interaction between the nanoparticles and the PP matrix is strong enough so as to restrict intermolecular sliding and subsequent yielding in the localized scale. Flexural strength and modulus of the nanocomposites were improved with increasing of the nano *α*-Al_2_O_3_ loading. Storage modulus and loss modulus of nano composites are improved with increasing the loading percentages of nano *α*-Al_2_O_3_. These observations show improvement in the stiffness and energy dissipation of the composites deformed under the periodic stress. The effect of dispersant (SDBS) on mechanical properties of PP/nano *α*-Al_2_O_3_ composites indicated that SDBS is comparable to a surfactant that breaks up the massive agglomerates into larger ones and supports the compatibility of nano *α*-Al_2_O_3_ with PP. Furthermore, TEM investigation is needed to gain full understanding of the dispersion of nanoparticles. With increasing of the nano *α*-Al_2_O_3_s content, the nanoparticle-rich areas become bigger and tend to show characteristics of forming an interconnecting network. When such network density becomes higher at higher nano *α*-Al_2_O_3_s concentrations, it can potentially lead to intense strain localization because of hindered plastic deformation of the matrix. The strain localization around the dense network of nano *α*-Al_2_O_3_s ultimately causes matrix cracking due to severe modulus mismatch between polymer and nanoparticles and hence ductile yielding behavior is substantially reduced.

Good and homogenous distribution of nano *α*-Al_2_O_3_ particles will provide good stress shift throughout mechanical loading which ensures better mechanical and morphological properties of the manufactured composites. From TEM micrographs, it was established that the presence of dispersant directly affected the size of the agglomerates.

As a conclusion, it can be stated that the PP/nano *α*-Al_2_O_3_ nanocomposites had an enormous potential to be considered in a lot of engineering requests, mainly for the purposes that need good strength, high stiffness and excellence toughness properties, and good physical and chemical resistance characteristics.

## Figures and Tables

**Table 1:** The weight content of the ingredients for PP/nano *α*-Al_2_O_3_ composites.

Nano *α*-Al_2_O_3_ content (wt%)	PP content (wt%)	TiO_2_ content (wt%)
0	100	0
1	98.98	0.02
2	97.96	0.04
3	96.94	0.06
4	95.92	0.08
5	94.9	0.1
